# Targeting Inflammatory Cytokines to Improve Type 2 Diabetes Control

**DOI:** 10.1155/2021/7297419

**Published:** 2021-09-13

**Authors:** Tsvetelina V. Velikova, Plamena P. Kabakchieva, Yavor S. Assyov, Tsvetoslav А. Georgiev

**Affiliations:** ^1^Department of Clinical Immunology, University Hospital “Lozenetz”, Sofia University “St. Kliment Ohridski”, Sofia 1407, Bulgaria; ^2^Clinic of Endocrinology, University Hospital “Alexandrovska, ” Department of Internal Medicine, Medical Faculty, Medical University of Sofia, Sofia 1431, Bulgaria; ^3^Clinic of Internal Medicine, Naval Hospital-Varna, Military Medical Academy, Varna 9010, Bulgaria; ^4^Clinic of Rheumatology, University Hospital “St. Marina, ” First Department of Internal Medicine, Medical Faculty, Medical University-Varna, Varna 9010, Bulgaria

## Abstract

Type 2 diabetes (T2D) is one of the most common chronic metabolic disorders in adulthood worldwide, whose pathophysiology includes an abnormal immune response accompanied by cytokine dysregulation and inflammation. As the T2D-related inflammation and its progression were associated with the balance between pro and anti-inflammatory cytokines, anticytokine treatments might represent an additional therapeutic option for T2D patients. This review focuses on existing evidence for antihyperglycemic properties of disease-modifying antirheumatic drugs (DMARDs) and anticytokine agents (anti-TNF-*α*, anti-interleukin-(IL-) 6, -IL-1, -IL-17, -IL-23, etc.). Emphasis is placed on their molecular mechanisms and on the biological rationale for clinical use. Finally, we briefly summarize the results from experimental model studies and promising clinical trials about the potential of anticytokine therapies in T2D, discussing the effects of these drugs on systemic and islet inflammation, beta-cell function, insulin secretion, and insulin sensitivity.

## 1. Introduction

Type 2 diabetes (T2D) is one of the most common chronic metabolic disorders in adulthood worldwide. It is characterized by relative insulin deficiency and dysfunction in glucose, lipid, and protein metabolism that further determine the common features of T2D such as hyperglycemia, dyslipidemia, metabolic dysfunction, and associated long-term macrovascular (atherosclerosis, coronary heart disease, cardiomyopathy, cerebrovascular disease, peripheral artery disease and lower extremity amputations) and microvascular complications (retinopathy, nephropathy, and neuropathy) [1].

Insulin resistance (IR) in target organs (liver, adipose tissue, skeletal muscles) and pancreatic beta-cell dysfunction has a fundamental role in the pathogenic mechanisms of T2D. IR is characterized by impaired insulin-mediated glucose uptake in target cells, and it is the most common driving feature presenting throughout the progression from prediabetes to overt T2D. In the pancreas, islet beta cells react to IR by enlarging their mass, which results in compensatory increased insulin secretion. About one-third of all obese people will develop T2D depending on the individual functional capacity of their islet beta cells to compensate for IR [2]. Furthermore, some T2D patients will gradually progress to overt insulin deficiency and will need insulin replacement therapy.

Still, the underlying mechanisms involved in the pathogenesis of IR and beta-cell dysfunction are poorly understood. The discovery of increased circulating inflammatory factors such as C-reactive protein (CRP), chemokines, and cytokines in T2D patients, as well as elevated levels of tumor necrosis factor-*α* (TNF-*α*) in the adipose tissue, associated with obesity-related IR and islet inflammation, creates a new era of understanding of the pathophysiology of T2D and sheds light on the pathogenic role of inflammation [3].

Obesity is one of the most significant risk factors for the development of T2D. Being a sizeable endocrine organ, the visceral adipose tissue is infiltrated with several types of immune cells like macrophages and T and B cells [4], as their number and inflammatory activity correlate with the degree of obesity and severity of IR [5, 6].

Infiltrated in the visceral adipose tissue, macrophages are the leading producers of the pro-inflammatory molecule TNF-*α* [2]. Moreover, in a state of overnutrition, visceral adipose tissue, liver, and pancreatic islets also secrete different inflammatory factors like interleukin-1*β* (IL-1*β*), interleukin-6 (IL-6), CRP, and various chemokines. Elevated levels of these inflammatory mediators reflect chronic low-grade tissue inflammation associated with obesity and could represent predictive factors for the development of T2D [7, 8].

Increasing evidence supports a pathogenic function for CD4+ T cells in obesity and IR. However, CD4+ T cells can contribute significantly to inflammation observed in obesity and obesity-related IR. Studies showed increased activation of CD4 + CD44^hi^CD62L^lo^ T cells in the visceral adipose tissue in obese mice [9] that express markers for cellular senescence (i.e., PD-1 and CD153) [10]. Obesity has also been found to increase the expression of major histocompatibility complex (MHC) class II molecules on adipocytes and to activate adipose CD4+ T lymphocyte-related inflammation. Additionally, because of the significant role of T cells in chronic inflammation and IR development, T2D cannot be currently considered as a typical autoimmune disease or classified as metabolic disorder only [11]. Therefore, it is necessary to develop innovative therapeutic strategies to modulate metabolic inflammation and IR. These innovative approaches depend on the understanding of the involvement of certain immune cells and proinflammatory mediators in obesity and T2D [12].

Additionally, obesity is a common risk factor for numerous rheumatic diseases (including rheumatoid arthritis, psoriatic arthritis, and ankylosing spondylitis) and is associated with more severe and treatment-refractory diseases [13]. Most inflammatory rheumatic conditions such as rheumatoid arthritis, systemic lupus erythematosus, Sjögren's syndrome, and psoriatic arthritis are regarded as autoimmune diseases, where single or more cells become self-antigens and are attacked by autoreactive T and B cells. Abnormal immune response and complement activation are also accompanied by cytokine dysregulation and inflammation [14]. Considerable evidence implicates tissue inflammation in pancreatic islet dysfunction, with local amyloid deposition and fibrosis leading to more severe islet inflammation [15]. A novel player in inflammation is interleukin-17 (IL-17), and several studies have already confirmed its role in the pathogenesis of various autoimmune and inflammatory disorders such as systemic psoriasis, rheumatoid arthritis, inflammatory bowel disease, systemic lupus erythematosus, multiple sclerosis [16], type 1 diabetes (T1D) [17], and even T2D [18].

**Figure 1:**
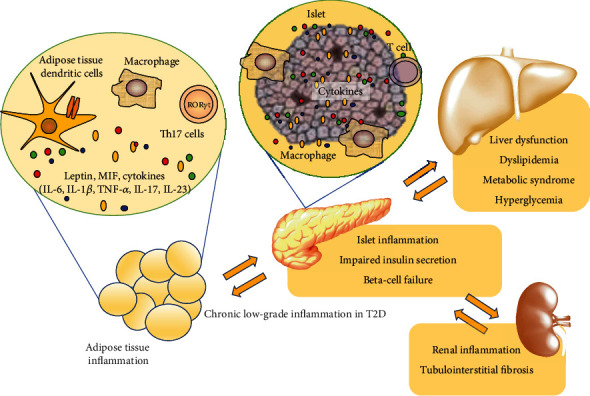
Chronic low-grade inflammation in type 2 diabetes in the pancreas, adipose tissue, liver, and kidney. Inflammatory responses include recruitment and activation of antigen-presenting cells such as dendritic cells and macrophages, different T cell subsets, secretion of proinflammatory cytokines and other mediators, and consequent impairment of beta-cell function, liver dysfunction, and renal damage. Abbreviations: Тh17: T-helper 17 cells; MIF: macrophage migration inhibitory factor; IL: interleukin; ROR*γ*t: retinoid-related orphan receptor gamma t; TNF-*α*: tumor necrosis factor-alpha.

The immune pathophysiology of T2D is presented in Figure 1. Expectedly, therapeutic strategies targeting inflammation in rheumatic diseases are focused on distinct targets of autoimmune response by using immunosuppressive drugs.

Promising results from clinical trials with interleukin-1 (IL-1) antagonists, TNF-*α* inhibitors, and blockade of IL-6 and IL-17 pathways [19–21] opened the door for anticytokine strategies in the treatment of T2D. This kind of treatment promises to synchronously improve subclinical inflammation, peripheral insulin sensitivity, and abnormal glucose homeostasis. This review focuses on existing evidence for antihyperglycemic properties of disease-modifying antirheumatic drugs (DMARDs) and anticytokine agents (anti-TNF-*α*, -IL-6, -IL-1, -IL-17, and -IL-23). Emphasis is placed on their molecular mechanisms of action and on the biological rationale for their clinical use. Finally, we briefly summarise results from encouraging clinical trials about the potential of anticytokine therapies in T2D, discussing the effects of these drugs on systemic and islet inflammation, beta-cell function, insulin secretion, and insulin sensitivity.

## 2. Search Strategy

To elucidate the potential role of inflammatory cytokines as targets for antidiabetic therapies, a narrative review was conducted in accordance with recently published recommendations for writing a narrative biomedical review [22]. Initially, we performed a systematic approach that implied grouping and analyzing sources by a thorough literature search in the bibliographic database MEDLINE. Both MeSH and relevant free-text terms were used in the following PubMed search: (“therapy” OR “treatment” AND (“diabetes”)) AND (“DMARDs” OR “biologics” OR “anti-cytokine”). Our search was limited to English language articles published until 30 January 2021. Finally, references of retrieved articles were further extracted and hand searched for relevant information. The synthesized qualitative data of experimental models and clinical trials of anticytokine treatments are summarized in Tables 1 and 2, respectively.

## 3. The Biological Rationale for Clinical Use of Anticytokine Therapies in T2D

Although inflammatory responses are implicated in the vigilant defense against infection and cancer, dysregulated or exuberant systemic inflammation plays a crucial role in the pathophysiology of numerous acute and chronic noninfectious conditions [23].

Together with beta-cell dysfunction, IR reflects an essential aspect of T2D pathophysiology. Recent findings suggest that both pathognomonic features of the disease are tightly related to innate and adaptive immunity [6]. Importantly, dysfunctional adipose tissue infiltrated with inflammatory cells contributes to IR and to excessive amounts of proinflammatory cytokines in patients with prediabetes and T2D [24, 25]. On the other hand, immune-mediated inflammation is deleterious to insulin secretion in beta cells [26]. Furthermore, a mounting body of evidence suggests T2D-related vascular and neurologic complications as inflammatory-driven processes finely tuned by the cytokine milieu [27]. This presumption provides the rationale for utilizing anticytokine therapies to manage patients with T2D [28].

Cytokines are a large family of cellular messenger molecules driving the inflammatory responses by regulating the balance between proinflammatory and anti-inflammatory signals. To date, various cytokines are targets for the biological treatment of autoinflammatory and autoimmune diseases, and anticytokine agents are broadly used as disease-modifying drugs in patients with rheumatic conditions. Additionally, some of these cytokines are involved in glucose metabolism as well [29].

IL-1 family cytokines are prototypical proinflammatory mediators and are therefore implicated in the treatment of a broad spectrum of inflammation-driven diseases. In the case of T2D, IL-1*β* may impair insulin secretion and mediate beta-cell apoptosis [30]. Additionally, IL-1 gene polymorphisms are linked to T2D susceptibility [31]. By inhibiting both IL-1*α* and IL-1*β* activity, the IL-1 receptor antagonist anakinra has been shown to improve metabolic dysfunction in patients with rheumatoid arthritis, leading to a significant reduction of glycated hemoglobin (HbA1c) [32]. Furthermore, patients with T2D alone display a significant improvement in glucose metabolism with IL-1 blockade, along with reduction in markers of systemic inflammation [33].

Similarly, IL-6 can affect glucose homeostasis and metabolism directly and indirectly by acting on skeletal muscle cells, adipocytes, hepatocytes, pancreatic beta cells, and neuroendocrine cells. Its primary immunological function is to potentiate the effects of other cytokines. Additionally, experimental investigations have shown that IL-6 is a predictor and pathogenesis-related biomarker for T2D development. Together with other proinflammatory cytokines, IL-6 can enhance beta-cell apoptosis [34].

IL-6 inhibition has already proven effective in rheumatoid arthritis with an acceptable safety profile [35]. Recent findings have drawn attention to expanding the therapeutic applications of IL-6 inhibition. In the case of T2D, IL-6 inhibition may have potential antidiabetic properties because IL-6 is known to contribute to IR by inducing the overexpression of SOCS-3 (suppressor of cytokine signaling 3), a possible inhibitor of insulin signaling. Furthermore, IL-6 impairs the phosphorylation of insulin receptor and insulin receptor substrate-1 [36]. Thus, IL-6 signaling may be a viable therapeutic target in T2D.

IL-17 is a family of inflammation-promoting cytokines that have been implicated in the pathogenesis of numerous autoimmune and immune-mediated diseases. Current evidence advocates the therapeutic blockade of IL-17 for the treatment of patients with plaque psoriasis, psoriatic arthritis, and ankylosing spondylitis. In addition, recent studies have demonstrated a close link between Th17 cells (a distinct CD4+ T-cell subset characterized by the ability to produce and secrete IL-17) and development of T2D [37]. Briefly, the IL-17 overexpression could decrease insulin sensitivity and contribute to IR by activating proinflammatory signaling pathways [11]. Indeed, newly diagnosed T2D patients have significantly higher levels of IL-17 in comparison with healthy controls [38]. Furthermore, in tight tandem with TNF-*α*, IL-17 may contribute to the inhibition of insulin signaling, resulting in IR by activating c-Jun N-terminal kinase [39].

IL-12 and IL-23 are heterodimeric cytokines secreted by activated antigen-presenting cells. They act as critical regulators of both innate and adaptive immunity. IL-23 plays a critical role in the activation of Th17 cells and in the production of IL-17 and other proinflammatory cytokines. At the same time, IL-12 stimulates natural killer (NK) cells and causes the differentiation of CD4 + T cells into the T helper 1 (Th1) phenotype, which elicits the Th1-mediated inflammatory pathway. Abnormal regulation of IL-12 and IL-23 has been associated with immune-mediated diseases such as psoriasis and psoriatic arthritis. For example, patients with T2D display increased pancreatic islet inflammation, initially characterized by local overexpression of inflammatory cytokines, including TNF-*α* and IL-12 derived from innate immune cells [40]. Furthermore, minor variants of IL-12B and IL-23R are associated with T2D in patients with plaque psoriasis and psoriatic arthritis [41].

TNF-*α* is defined as a pleiotropic cytokine due to its ability to act on different cell types. Studies have already proven its role in noninfectious processes such as angiogenesis, morphogenesis, metastasis, allergy, and glucose metabolism [42]. In addition, TNF-*α* has pronounced proinflammatory properties. It is one of the cytokines that initiate the inflammatory cascade and is, therefore, often referred to as the “primary cytokine.” TNF-*α* exhibits its proinflammatory properties by activating the nuclear transcription factor kappa B (NF-*κ*B). TNF-*α* is produced by adipocytes and inflammatory cells in response to chronic inflammation, and its serum levels are strictly related to obesity in T2D [43]. Consequently, the raised levels of TNF-*α* in obese people induces IR by disrupting insulin signaling through serine phosphorylation of insulin receptor substrate 1 [44]. The pleiotropic nature of TNF-*α* defines a broad potential for the treatment of autoimmune and immune-mediated disorders such as rheumatoid arthritis, ankylosing spondylitis, psoriasis, inflammatory bowel disease, refractory asthma, and hidradenitis suppurativa. Strong evidence already supported that modulating the TNF-*α* pathway in patients with inflammatory rheumatic conditions and T2D could improve insulin sensitivity [45] and modestly lower HbA1c values [46].

To sum up, a shred of mounting evidence has recently shown that immune-mediated mechanisms drive T2D onset and development. Thus, targeting chronic low-grade inflammation may provide a rationale for the clinical use of anticytokine agents and DMARDs in subjects with T2D and related vascular and neurologic complications, which are inflammatory-driven processes finely tuned by cytokine cross-talk.

## 4. In Vitro and In Vivo Studies on Anticytokine Treatments in T2D

As the inflammation was associated with T2D and its progression with the balance between pro and anti-inflammatory cytokines, anticytokine agents might represent an additional therapy option. Therefore, different anticytokine agents have been investigated in experimental models of T2D to assess their effects on systemic and islet inflammation, beta-cell function, insulin resistance, and glucose homeostasis [28].

Recent findings showed the crucial role of IL-17 and Th17 cells in the inflammatory process and development of T2D [39]. Various preclinical studies have proven the involvement of Th17 cells and IL-17A in the pancreas injury observed in diabetes [47]. Studies revealed that treatment with anti-IL-17 neutralizing antibodies increased serum levels of adiponectin (an insulin-sensitizing adipokine), promoted adipocyte differentiation, and decreased serum TNF-*α* levels. These findings suggested that IL-17 may have a critical role in IR and T2D development [48]. It is well known that IL-17 activates NF-*κ*B that upregulates the expression of other inflammatory cytokine genes [49]. Thereby, IL-17, by increasing the expression of proinflammatory cytokines (i.e., IL-1*β*, IL-6, and TNF-*α*), eventually leads to the development of IR and T2D [39].

In line with this, the administration of drugs that target IL-17 or directly block Th17 cells can control autoimmune diabetes in NOD (nonobese diabetic) mice, and this has previously been suggested as a therapeutic strategy for T1D. Blocking IL-17 activity decreases peri-islet T cells and IL-17 concentrations, showing a substantial effect on diabetes prevention in NOD mice [50]. Other preclinical investigations have shown the benefits of blocking Th17/IL-17A in NOD mice treated with a selective inverse agonist of retinoic acid-related orphan receptor *α*/*β*, a key transcription regulator of Th17 cell differentiation. The results showed a significantly reduced incidence of diabetes, along with reduced insulitis and proinflammatory cytokine expression [51].

Although these studies obtained data from animal models of T1D, by considering the role of the Th17 cells pathway in T2D, we can speculate that this therapy could be implemented with great benefits for T2D patients. However, there are some controversial data on IL-17 blockade in T2D. Conflicting evidence on the IL-17A protective function on diabetes incidence were documented in experimental diabetic nephropathy (DN) [52]. Interestingly, the renal lesions were more severe in IL-17A-deficient mice than in wild mice [53]. This is surprising because targeting IL-17A was demonstrated to lower diabetes severity. In addition, an experimental model of T1D used in the same research (Ins2Akita mice) showed that treatment with low dosage recombinant IL-17A or IL-17F decreased albuminuria and renal damage [53]. While it was suggested that the reduction of STAT-3 (signal transducer and activator of transcription-3) activation might credit the protective effects of IL-17A and IL-17F, recent research on streptozotocin- (STZ-) induced diabetic models, on the other hand, revealed opposing results. Conversely, the protective effects of IL-17A monoclonal antibody therapy were decreased in IL-17A knock-out mice, which are insufficient in IL-17 compared to the wild-type mice. In conclusion, the administration of an anti-IL-17 monoclonal antibody to diabetic wild-type mice was protective, and the renal lesions were thus decreased [54].

Nevertheless, it was suggested that IL-17A might promote DN, especially in T2D models using db/db mice (carriers of leptin receptor gene mutation), which are outstanding examples for early DN studies [39]. Recently, Lavoz et al. demonstrated the reversal of structural abnormalities in DN (including improvement of mesangial matrix accumulation, mitigation of renal inflammation and improvement of renal function) after the administration of neutralizing anti-IL-17A antibodies to BTBR ob/ob mice (carrying leptin deficiency mutation) [47]. In leptin-deficient BTBR ob/ob mice, the favorable effects of an anti-IL-17A neutralizing antibody, including inhibition of both NF-*κ*B activation and overexpression of related genes (i.e., monocyte chemoattractant protein-1 or MCP-1), were linked with the suppression of inflammatory pathways. This pathway was independent of glycemic control [47]. Activation of the IL-17A/NF-*κ*B pathway, therefore, leads to renal injury related to diabetes. The same authors have previously demonstrated the dramatically increased expression of kidney MCP-1 and RANTES (regulated upon activation, normal T cell expressed and presumably secreted) gene after injection of IL-17A in C57BL/6 mice, which resulted in the attraction of inflammatory cells to the kidney [55]. In addition, inhibition of IL-17A also reduced proinflammatory gene overexpression and inflammatory cell infiltration in an experimental angiotensin II infusion model in mice [56]. These results indicate that high local IL-17A production in diabetic kidneys can further trigger resident renal cells to generate proinflammatory cytokines, such as MCP-1 and chemokines, to further attract inflammatory cells into the diabetic kidney [56]. Another study conducted by Qiu et al. revealed that anti-IL-17A treatment alleviates diabetic retinopathy in rodents [57]. These observations suggest that IL-17A blockade may be a feasible therapeutic option for some of the most common T2D complications, such as DN. However, the translation of such findings into clinical settings is still lacking.

Additionally, Lee et al. showed that anti-IL-17A and anti-IL-23 blocking antibodies in obese diabetic mice enhanced the reepithelization of skin wounds. They found that IL-23- but not IL-12-deficient mice displayed significantly reduced IL-17 expression in skin wounds. However, this was reversed by the delivery of recombinant IL-23. Therefore, when treated with anti-IL-17A and anti-IL-23p19 blocking antibodies, obese diabetic mice had significantly accelerated wound healing. Similarly, IL-17^−/−^ obese mice had improved wound re-epithelialization [58].

Furthermore, it was shown that the improvement in insulin sensitivity at the molecular level was linked to the restoration of the insulin signaling cascade during anti-TNF-*α* treatment [20]. Then, in the case of obese nondiabetic subjects, TNF-*α* inhibition was observed to dramatically decrease fasting glucose levels and to increase total adiponectin concentrations; however, these changes were accompanied by increased skeletal muscle adiposity [59, 60]. All these observations of TNF-*α*inhibitors in T2D patients should be confirmed in prospective clinical trials.

Given the crucial role of IL-1*β* in beta-cell dysfunction, numerous studies conducted on animal models of T2D have evaluated the effect of IL-1*β* neutralization on glucose tolerance. Treatment with IL-1 receptor antagonist (IL-1Ra) has led to reduced immune cell infiltration into the pancreatic islets of GK rats (a spontaneous, nonobese model of T2D). The metabolic effects included improvements in insulin secretion and glycemic control [61].

In a rat model of islet amyloidosis, therapy with IL-1Ra also improved beta-cell function and reduced insulitis [62]. Moreover, anti-IL-1*β* antibody treatment reduced islet infiltration and beta-cell death and improved insulin secretion and glucose control in mice fed a high-fat diet [63]. In addition, the recombinant IL-1Ra anakinra has been shown to partially reverse beta-cell dysfunction in glucotoxicity- and lipotoxicity-induced injury in human islet cultures [64, 65].

Future perspectives include treating T2D with designer cytokines, such as that studied by Findeisen et al., who engineered the gp130 ligand IC7Fc. This is a cytokine associated with a ciliary neurotrophic factor- (CNTF-) like IL-6-receptor-dependent signaling [66]. The investigators showed that IC7Fc improved glucose tolerance and hyperglycemia by increasing plasma levels of insulin and C-peptide in both high-fat diet and leptin receptor deficient mouse models. Additionally, alleviation of hepatic steatosis and weight gain was documented in mice. Besides, through activating the YAP1 (yes-associated protein 1) transcriptional regulator, skeletal muscle mass was preserved [66]. IC7Fc therapy does not cause inflammation or immunogenicity in human cell-based tests or other nonhuman models that mimic clinical trials (i.e., long-tailed macaques). Therefore, IC7Fc is a viable biological agent for potential future applications in the treatment of disorders such as T2D and muscle atrophy, thanks to its excellent safety profile and desired effects. In general, these findings support IC7Fc's further development as a biological treatment for T2D, ready to be included in phase I trials [66].

The published and available findings coming from experimental studies on anticytokine agents used for treatment of T2D are presented in Table 1.

## 5. Clinical Trials of Anticytokine Treatments for T2D

Many observational and short-term intervention trials showed the potential of DMARDs to improve glucose management and to lower the risk of acquiring diabetes in patients with inflammatory rheumatic diseases, with and without T2D, respectively. However, there is a dearth of long-term safety evidence for these medications in diabetic patients regarding their long-term usage. A chronic inflammation linked to obesity was linked to T2D and cardiovascular conditions associated with atherosclerotic diseases. In addition, inflammatory mediators are often associated with decreased beta-cell function and poor glucose control [67]. An increasing number of preclinical and clinical studies supported the inflammation theory in T2D. However, clinical trials evaluating the use of anti-inflammatory agents (such as anti-cytokine agents) are currently underway to verify the potential of these agents in the prevention and treatment of T2D [68].

Previous clinical studies have consistently shown that the impairment of glucose homeostasis is tightly related to inflammatory activity in patients with rheumatoid arthritis [69, 70]. It is, therefore, plausible that targeting the inflammatory state could improve glucose abnormalities in such patients. However, only a few anti-inflammatory molecules have been tested in clinical settings to treat T2D. These include salsalate, salicylic acid derivatives, and specific anti-cytokine agents such as IL-1 antagonists and TNF-*α* inhibitors [68].

Several antidiabetic medications, lipid-lowering drugs, and antihypertensive medications also have anti-inflammatory properties, such as insulin, incretins, statins, and angiotensin-converting enzyme inhibitors [71]. Recently, Infante and colleagues reviewed the antihyperglycemic properties of the DMARD hydroxychloroquine in patients with diabetes [72]. The usefulness of anti-IL-1*β* treatment in patients with T2D has also been investigated. A randomized clinical trial of anakinra, a recombinant human IL-1 receptor antagonist, has significantly ameliorated markers of systemic inflammation, HbA1c values, glycemia, and beta-cell function in patients with T2D [33]. Furthermore, other smaller studies have also shown benefits on HbA1c values following treatment with other anti-IL-1 therapies [73, 74]. A recent meta-analysis of 2921 individuals from eight phase I-IV studies showed that IL-1 antagonism was associated with a significant overall HbA1c-lowering effect; in addition, meta-regression analyses revealed a significant correlation between baseline CRP and C-peptide and HbA1c outcome [21]. However, in a large randomized clinical trial conducted in over 4000 subjects with prior myocardial infarction, treatment with canakinumab (a human monoclonal antibody targeting IL-1*β*) for a period of 3.7 years did not reduce the risk for incident diabetes [19].

Emerging evidence also suggests that anti-IL-1*β* therapy may dramatically decrease the risk of macrovascular and microvascular complications of diabetes [19, 75, 76]. Other studies using IL-1*β* inhibition [77–79], and the IL-1 receptor antagonistanakinra [80, 81] demonstrated similar outcomes, including increased insulin secretion and reduced HbA1c values. In contrast, Ridker et al. [82] did not find alterations in HbA1c, glucose, and insulin levels after canakinumab treatment in well-controlled T2D patients with high cardiovascular risk.

Clinical studies on the impact of TNF-*α* inhibitors on glucose control and insulin sensitivity are also scarce. In theory, blocking TNF-*α* may improve insulin sensitivity by increasing the tyrosine kinase activity of the insulin receptor and thus could promote glucose uptake in peripheral tissues [83]. A randomized, placebo-controlled study conducted in 40 obese individuals with metabolic syndrome has shown a significant reduction in fasting glucose levels and an increased ratio of high molecular weight adiponectin to total adiponectin following a 6-month treatment course with the TNF inhibitor etanercept [59]. Furthermore, targeting TNF-*α* in patients with rheumatoid arthritis may ameliorate the phosphorylation of the insulin signaling cascade [20]. A meta-analysis of eight studies involving 260 subjects has shown that TNF-*α* inhibition leads to a significant reduction in HOMA (Homeostatic Model Assessment for Insulin resistance) and QUICKI (Quantitative Insulin Sensitivity Check Index) indices, which are surrogate markers for IR [84]. The study included patients with rheumatoid arthritis, psoriasis, ankylosing spondylitis, and inflammatory bowel disease, in whom neutralizing TNF-*α* (with drugs such as infliximab or etanercept) exerted beneficial effects on IR and risk of T2D development. It was also shown that TNF-*α* inhibition might delay the progression of autoimmune diseases by reducing inflammation [84].

Conversely, infliximab did not alter insulin secretion in patients with Crohn's disease [85]. Furthermore, a double-blind study conducted in patients with obesity and T2D showed that treatment with TNF-*α* did not improve fasting insulin, glucose, and C-peptide over a period of 4 weeks [86]. Other clinical trials involving patients with metabolic syndrome, IR, and T2D on anti-TNF-*α* agents demonstrated improved insulin sensitivity, reduced HbA1c values, and increased adiponectin levels [60, 87], or no effect on insulin sensitivity [88–90].

Since the IL-23-Th17 pathway has been discovered, many research studies have been conducted on this new treatment strategy, with optimistic outcomes for autoimmune disorders such as rheumatoid arthritis and T1D [91, 92].

Future therapeutics and therapeutic strategies will focus on Th17 cells since these cells have been shown to play an important role in both T1D and T2D. Therefore, therapeutic strategies should be focused on Th17 cells rather than on specific Th17-related cytokines. However, IL-23 also plays a crucial role in driving the production of IL-17 by maintaining Th17 cells [93].

It was shown that anti-IL-17 treatment in T2D increased serum adiponectin levels while reducing serum TNF-*α* concentration. Additionally, adipocyte differentiation markers were upregulated. Thus, therapy with antibodies blocking IL-17 improves adipose tissue functionality, resulting in the release of the anti-inflammatory and insulin-sensitizing adipokine adiponectin [48]. Such antibodies appear to be safe and well tolerated for treatment in different clinical settings. Whether targeting IL-17 and Th17 cells can protect pancreatic islets in children with islet autoimmunity should be clarified in future studies [50]. Moreover, the use of anticytokine therapy for DN or lupus nephritis has not been studied yet. Given the vast amount of information collected lately on the role of inflammation in DN, future studies involving the use of anti-IL-17A antibodies in DN are warranted [94].

Clinical outcomes of the most relevant clinical trials of anticytokine therapies in patients with T2D are summarized in Table 2.

Nevertheless, anticytokine therapies are not devoid of side effects; thus, benefits should be carefully weighed against potential harms related to the use of these drugs. Critical adverse events deriving from the use of these drugs (ranging across different biological agents) include infections (including reactivation of latent tuberculosis and hepatitis B), rare demyelinating disorders of the central nervous system, and liver and cardiac injury [95, 96]. Therefore, these aspects need further investigation in future studies.

## 6. Conclusions

Mounting evidence has recently elucidated some of the immune-mediated mechanisms that drive T2D onset and development. Given the role of interleukin pathways in T2D-related inflammation, different anticytokine agents have been investigated in experimental models of T2D, and clinical trials have been conducted or are underway to assess the effects of these agents on systemic and islet inflammation, beta-cell function, insulin resistance, and overall glucose control. Targeting chronic low-grade inflammation may provide a rationale for the use of anticytokine agents and DMARDs for prevention and/or treatment of T2D and its related microvascular and macrovascular complications, which are inflammatory-driven processes finely tuned by the cytokine crosstalk. Indeed, an increasing number of preclinical and clinical investigations support the “inflammation theory” in T2D. However, there is a need for prospective long-term investigations of these drugs in diabetic patients, which are warranted particularly to address the potential safety issues deriving from their long-term use.

## Figures and Tables

**Table 1:** Representative experimental studies on anticytokine agents for treatment of type 2 diabetes (T2D).

Author, reference	Model	Drug	Main findings
Maedler et al., 2004 [65]	Glucotoxicity- and lipotoxicity-induced injury in human beta cells	Recombinant IL-1 receptor antagonist (anakinra)	Increased levels of insulin, protection against glucose-induced beta-cell apoptosis
Ehses et al., 2009 [61]	T2D GK rats	IL-1 receptor antagonist	Reduced islet immune cell infiltration, decreased glucose levels, increased insulin levels
Böni-Schnetzler et al., 2009 [64]	Human islet cultures from T2D patients	Recombinant IL-1 receptor antagonist (anakinra)	Reversal of beta-cell dysfunction
Westwell-Roper et al., 2015 [62]	Lean and obese male mice	IL-1 receptor antagonist	Improved glucose tolerance and reduced plasma proinsulin: insulin ratio, no effect on insulin sensitivity, reduced severity, and prevalence of islet amyloid deposition
Sauter et al., 2015 [63]	C57BL/6N mice	Anti-IL-1*β* antibody	Reduced beta-cell apoptosis, restored insulin secretion, and improved glycemia
Solt et al., 2015 [51]	NOD mice	Selective inverse agonist of ROR*α*/*β*	Reduced incidence of diabetes, reduced insulitis, and proinflammatory cytokine expression
Mohamed et al., 2016 [53]	STZ-induced-diabetic mice, db/db mice and Ins2Akita mutant mice	Low-dose IL-17A administration	Decreased albuminuria and renal damage
Qiu et al., 2017 [57]	High glucose (HG)-treated rat Müller cell line (rMC-1)	Anti-IL-17A or anti-IL-17RA	Improved retinal Müller cell dysfunction, reduced vascular leukostasis and vascular leakage, reduced tight junction protein downregulation, and ganglion cell apoptosis in the retina
Lee et al., 2018 [58]	Obese diabetic mouse wounds	Anti-IL-17 and anti-IL-23 blocking antibodies	Accelerated diabetic wound healing through alteration of macrophage polarization
Ma et al., 2019 [54]	Streptozotocin-induced diabetic nephropathy through IL-17 knockout mice	Monoclonal anti-IL-17 antibody	Prevention of progression of diabetic nephropathy, reduced albuminuria, glomerular damage, macrophage accumulation, and renal fibrosis
Lavoz et al., 2019 [47]	BTBR Ob/Ob (leptin deficiency mutation) mice with diabetic nephropathy	IL-17A neutralizing antibody	Ameliorated renal dysfunction and reduced disease progression
Orejudo et al., 2019 [55]	C57BL/6 mice	IL17A neutralizing antibody	Reduced kidney inflammatory cell infiltrates and chemokine overexpression
Findeisen et al., 2019 [66]	Mice fed a high-fat diet and leptin receptor deficient mice models	gp130 ligand IC7Fc	Improved glucose tolerance and hyperglycemia by increase in plasma insulin and C-peptide levels, preserved skeletal muscle mass

**Table 2:** Summary of the most representative clinical trials of anticytokine therapies in patients with insulin resistance (IR), type 2 diabetes (T2D), and/or other metabolic dysfunctions.

Author, reference	Type of study and study duration	Drug	Patients/subjects	Main findings
Ofei et al., 1996 [86]	Double-blind clinical trial (8 weeks)	Engineered human anti-TNF-*α* antibody (CDP571)	Obese patients with T2D	No effect on insulin sensitivity
Paquot et al., 2000 [88]	Single-center, single-blind, sequential treatment (placebo, followed by active drug) study (6 days)	TNF-*α* antagonist: Ro 45-2081 (a recombinant fusion protein that consists of the soluble TNF-receptor p55 linked to the fc portion of human IgG1); single intravenous administration	Obese insulin-resistant patients	No effect on insulin sensitivity
Kiortsis et al., 2005 [87]	Clinical prospective study (6 months)	Anti-TNF-*α* (infliximab)	Insulin-resistant patients with rheumatoid arthritis and ankylosing spondylitis	In the entire study group, no significant changes of the HOMA index or QUICKI were observed. In the tertile of patients with the highest degree of insulin resistance, a significant decrease of the HOMA index and increase of the QUICKI was found
Dominguez et al., 2005 [89]	Parallel-group open-label randomized trial (4 weeks)	Anti-TNF-*α* (etanercept)	Obese patients with T2D	No effect on insulin sensitivity, increased insulin secretion
Bernstein et al., 2006 [90]	Double-blind, randomized controlled trial (4 weeks)	Anti-TNF-*α* (etanercept)	Patients with metabolic syndrome	No effect on insulin sensitivity, increased adiponectin levels
Lo et al., 2007 [60]	Randomized controlled trial (4 weeks)	Anti-TNF-*α* (etanercept)	Patients with metabolic syndrome	Decrease in glucose levels and HbA1c values, increased adiponectin levels
Larsen et al., 2007 [33]	Double-blind, parallel-group, randomized controlled trial (13 weeks)	Anti-IL-1 receptor antagonist (anakinra)	Patients with T2D	Reduced HbA1c values, increased insulin secretion, improved glycemia and beta-cell secretory function
Larsen et al., 2009 [80]	Double-blind, 39-week follow-up study	Anti-IL-1 receptor antagonist (anakinra)	Patients with T2D	Increased insulin secretion, decreased insulin requirements
van Asseldonk et al., 2011 [81]	Randomized, placebo-controlled trial (4 weeks)	Anti-IL-1 receptor antagonist (anakinra)	Nondiabetic, obese subjects with metabolic syndrome	Increased disposition index, improved insulin secretion, no effect on insulin sensitivity
Stanley et al., 2011 [59]	Randomized controlled trial (6 months)	Anti-TNF-*α* (etanercept)	Obese subjects with features of the metabolic syndrome	Decreased fasting glucose levels and HbA1c values, increased ratio of high molecular weight adiponectin to total adiponectin
Cavelti-Weder et al., 2012 [73]	Randomized, placebo-controlled trial (3 months)	Anti-IL-1*β* (gevokizumab)	Patients with T2D	Reduced HbA1c values, increased insulin secretion
Rissanen et al., 2012 [77]	Randomized, placebo-controlled trial (4 weeks)	Anti-IL-1*β* (canakinumab)	Patients with T2D and impaired glucose tolerance	Trend towards increased insulin secretion rate
Ridker et al., 2012 [82]	Randomized, placebo-controlled multinational phase IIb trial (5 months)	Anti-IL-1*β* (canakinumab)	Well-controlled T2D patients with high cardiovascular risk	No changes in HbA1c values, glucose, and insulin levels
Stagakis et al., 2012 [20]	Clinical prospective study (12 weeks)	Anti-TNF-*α* agents (infliximab, adalimumab, etanercept)	Patients with rheumatoid arthritis	Anti-TNF therapy improved insulin sensitivity and reversed defects in the insulin signaling cascade in patients with active disease and high insulin resistance
Hensen et al., 2013 [78]	Randomized, placebo-controlled trial (4 months)	Anti-IL-1*β* (canakinumab)	Patients with T2D	Reduced HbA1c values, nonsignificant increase in insulin secretion
Sloan-Lancaster et al., 2013 [74]	Phase II, randomized, double-blind, parallel, placebo-controlled study (12 weeks)	Neutralizing IL-1*β* antibody (LY2189102)	Patients with T2D	LY2189102 modestly reduced HbA1c values and fasting glucose levels
van Popper et al., 2014 [79]	Randomized, double-blind, placebo-controlled, crossover study (8 weeks)	Anti-IL-1 receptor antagonist (anakinra)	Subjects with impaired glucose tolerance	Improved insulin secretion (first-phase insulin secretion), improved insulinogenic index
Burska et al., 2015 [84]	Systematic review and meta-analysis	TNF-*α* inhibitors	Patients with rheumatoid arthritis	TNF inhibition therapy improved insulin sensitivity and reduced insulin resistance
Everett et al., 2018 [19]	Randomized, double-blind, placebo-controlled trial (3 months)	Anti-IL-1*β* (canakinumab)	Subjects with T2D, prediabetes and normal glucose tolerance	Canakinumab reduced HbA1c values during the first 6 to 9 months of treatment, but no consistent long-term benefits on HbA1c or fasting plasma glucose were observed
Kataria et al., 2019 [21]	Meta-analysis of 2921 individuals from eight phase I-IV studies	Anti-IL-1 therapies	Patients with T2D	Reduced HbA1c values
